# Person-First or Identity-First: Language Preferences and Effects on Social Perceptions of Disability

**DOI:** 10.3390/bs16101865

**Published:** 2026-10-09

**Authors:** Sidney K. Spilfogel, Lucas J. Teunissen, Stuart B. Kamenetsky

**Affiliations:** 1 Department of Psychology, University of Toronto, Toronto, ON M5S 3G3, Canada; sidney.spilfogel@mail.utoronto.ca (S.K.S.); lucas.teunissen@mail.utoronto.ca (L.J.T.); 2 Department of Psychological and Brain Sciences, University of Toronto Mississauga, Mississauga, ON L5L 1C6, Canada; 3 Institute for the Study of University Pedagogy, University of Toronto Mississauga, Mississauga, ON L5L 1C6, Canada

**Keywords:** disability, disability language, person-first language, identity-first language, identity perception, identity centrality, language preferences

## Abstract

Disability language use shifts with changing social attitudes. While person-first language (PFL) emphasizes humanity, identity-first language (IFL) depicts disability as integral to one’s identity. This study of 238 participants aged 18–30 investigated language preferences and whether language type influences perceptions of identity centrality. Participants rated the importance of identity markers to a PFL- or IFL-framed subject and indicated whether PFL or IFL are preferable in conversation. Results show that perceptions are influenced by identity type (i.e., disability versus non-disability), but not by whether identity is framed with PFL or IFL. PFL is preferred for most disabilities, except for sensory disabilities, for which, along with non-disability-related identities (e.g., race), IFL is preferred. These findings suggest that, despite current social trends favouring PFL, PFL versus IFL framing may have little effect on perceptions of people with disability. Dispositional factors were not found to be strong predictors of language preference, suggesting that disability language and the conceptualization of disability may be shaped by social factors rather than by internal states. Thus, considering the preferences and experiences of disability communities when referencing disability may foster more meaningful change in how disability is conceptualized.

## 1. Introduction

Language, as a human phenomenon, sways with the ebb and flow of sociocultural changes, reflecting what society-at-large values and deems important to communicate (Andrews et al., 2022; Haller et al., 2006). As a result, the labels used to describe disability have evolved with society’s views and attitudes (Andrews et al., 2022; Dunn & Andrews, 2015). The moral model—which views people with disabilities (PWDs) as unholy, in need, and objects of pity—dominated for much of human history, accompanied by derogatory labels that socially and culturally isolated them (Andrews, 2017). Following the Scientific and Industrial Revolutions, disability is now better understood through a biological lens, birthing the medical model of disability (Andrews, 2017; Dunn & Andrews, 2015; Smart, 2009). This model, while promoting scientifically based treatments for biological deficiencies, developed descriptions and labels for PWDs based on their disability, depersonalizing and pathologizing the individual and spotlighting their impairments at the forefront of their identity (Andrews, 2017; Dunn & Andrews, 2015; Smart, 2009).

The 1960s US Civil Rights Movement brought about inclusivity as an ideology—in education, employment, housing, and social policy, among other areas—and language quickly trailed the contemporary shift in social values (Andrews, 2017; Dunn & Andrews, 2015; Kaplan, 2000). Person-first language (PFL), in which an individual’s disability comes second to their status as a human being (e.g., person with a disability; Andrews et al., 2022), aimed to reduce negative perceptions of PWDs by stopping a “spread effect” where negative judgements of impairment lead to global negative evaluations of the individual (Andrews et al., 2022; Wright, 1964). As a result, PFL gained widespread popularity in common language and became the “correct” way to reference disability (Andrews et al., 2022; Wright, 1964). PFL became standard, encouraged by academic journals and inclusive language guidelines, such as the American Psychological Association (APA) bias-free language guidelines and the Government of Canada’s guide for communicating with and about PWDs (Andrews et al., 2022; APA, 2024; Gernsbacher, 2017; Government of Canada, 2024; Haller et al., 2006; Kapitan, 2017; Ladau, 2015).

This shift in language use paralleled a shift in disability models; the social model of disability, viewing disability as a socially constructed disadvantage failing to accommodate diverse physical and psychological needs, dissuaded the exclusion and inequity PWDs faced by encouraging changes in social systems and structures, including language (Andrews, 2017; Haller et al., 2006; Kaplan, 2000).

While there is major sociomoral and ideological backing for the use of PFL, linguists provided further academic support. The Sapir-Whorf hypothesis (SWH) is a long-standing theory in the field of psycholinguistics, broadly positing that language shapes the way people think (Sapir, 1929; Whorf, 1956). Within social cognition, the SWH suggests that linguistic structures not only influence individual thought, but also guide how people perceive and categorize others, shaping social judgments and identity-related inferences (Kuang & Bicchieri, 2024; Semin, 2001; Sutton, 2010). For disability language, PFL is often emphasized because, by placing “person” at the front of a label, it shifts attention toward a PWD’s humanity as opposed to their disability (Kapitan, 2017). However, the SWH is a divisive area of research with nuance surrounding its claims, such as the cognitive depth at which language influences thought (Wolff & Holmes, 2010). Nonetheless, the framework of the SWH serves as a theoretical motivation to investigate the effects of linguistic framing on disability and on identity-related cognition.

While PFL is pedestalled as the social standard for disability language, there has been pushback against its use from the disabled community (Andrews et al., 2022; Brown, 2011; Dunn & Andrews, 2015; National Federation of the Blind, 1993; Smart, 2009). Groups such as the Deaf, blind, and autistic communities advocate for identity-first language (IFL), in which an individual’s disability is mentioned first when referring to them (e.g., disabled person). IFL is based in the sociopolitical model of disability, rooted in civil rights and centres around the rejection of ableism, fostering self-acceptance, and an emphasis on disability being central to one’s identity. Advocates for IFL argue that this is the language structure used to refer to other groups (e.g., racial identity), and the attempt to separate disability from identity that occurs with PFL reinforces the notion that disability is an undesirable characteristic (Andrews et al., 2013, 2022; Dunn & Andrews, 2015). With support for both PFL and IFL, Dunn and Andrews (2015) encourage asking disabled individuals what language type they prefer and to use this as a guideline since preferred language shifts between and within individuals with various disabilities (APA, 2024).

While opinions and attitudes on whether PFL or IFL is most appropriate vary, research on the syntactic construction of phrases introduces further controversy in how the inherent linguistic differences in disability language types, beyond social and ideological preferences, engender different attitudes toward PWDs. Syntactically, PFL and IFL differ in their word order; in PFL (e.g., person who is blind), the head noun “person” precedes its adjunctive modifier “blind,” and in IFL (e.g., blind person), the modifier precedes the head noun. This makes IFL a pre-modified noun phrase (NP) and PFL a post-modified NP. The effects of modifier-noun order on cognition were first investigated by Karimi et al. (2019) by looking at differences in encoding and retrieval for pre-modified and post-modified NPs. Their study found enhanced encoding and retrieval for post-modified NPs. Weber (2024) further assessed how modifier position impacts the perceptual qualities encoded in the modifier being attributed to the head noun, finding no significant results for the enhancement of perceptual qualities between pre- or post-modified NPs. Thus, a linguistic perspective provides a more structural and systematic analysis in comparing the differences between disability language types and investigating how preferences and attitudes are shaped by linguistic elements.

Earlier research on disability language use primarily focused on how presenting information using IFL or PFL influenced attitudes towards PWDs (Granello & Gibbs, 2016; Kamenetsky & Sadowski, 2020; Kelly et al., 2010; Patterson & Witten, 1987). These studies yielded mixed results, indicating that language type has no impact on attitudes (Kamenetsky & Sadowski, 2020; Patterson & Witten, 1987). However, some studies found PFL to evoke more positive attitudes and IFL more restrictive ones (Granello & Gibbs, 2016; Kelly et al., 2010). Despite these results, many professional workplaces and healthcare services strictly enforce PFL, suggesting some stigma or negative attitudes associated with IFL (Andrews et al., 2022; Brown, 2011; Dunn & Andrews, 2015).

Lynch et al. (1994) assessed individuals’ preference for language type during conversation by asking participants to choose which phrase they would use (IFL or PFL or no preference). They found that PFL was generally preferred by participants; however, language preferences were influenced by disability type. When referring to a “person with quadriplegia” versus “quadriplegic,” preferences were 36% to 33% respectively, with 23% describing no preference, while for “person with a disability” versus “disabled person,” preferences were 60% to 14%, with 26% describing no preference. Language preference may shift with disability type due to disabilities being associated with different stereotypes and beliefs. Timmons et al. (2023) and Canton et al. (2023) found that the level of ableism and levels of perceived warmth, competence, and courage vary across disability types.

Beyond the influence of disability type, disability identity itself is often viewed as a distinguishing identity marker that creates conceptual separation between disability and non-disability. Marks (1999) describes disability as often socially categorized as distinct due to perceived differences in functional capacity, as well as indicators that signal physical or cognitive exceptionalities. As such, PWDs have certain shared experiences that result from social and institutional labels that deem disability as categorically distinct (Haller et al., 2006; Marks, 1999). Murugami (2009) describes a psychological tension commonly seen in disability experiences; a core difficulty for PWDs’ self-concept formation is the breadth of the impact of disability in personal development, including daily decision-making, vocational and educational experiences, and community integration. Murugami’s (2009) focus on self-concept suggests that differences between disabled and non-disabled experiences may be intertwined with broader social and cultural processes, e.g., language, which highlight disability as a prominent component of identity. Furthermore, disability may be conceptualized differently from other identity markers, such as race or sexual orientation, due to impact on functional capacity, supporting further investigation into how language such as PFL or IFL plays a role in shaping the disability identity as different from non-disability identities.

Beyond social influences on disability language and identity, prior research suggests that internal disposition or attributes also play a role in influencing language patterns (Kim et al., 1996; Muasya, 2023). Kim et al. (1996) compared what individualistic and collectivistic cultures deem most valuable during communication based on differences in self-construal—how people view themselves in relation to those around them (Markus & Kitayama, 1991). An independent self-construal, more common in individualistic cultures, views the self as distinct from others, typically with a stable personal identity that minimally varies with social context. On the other hand, an interdependent self-construal, more common in collectivistic cultures, prioritizes group harmony while the self is viewed as more flexible, context-dependent, and linked to the people around them. Kim et al. (1996) found that individuals with a more independent self-construal rated clarity and effectiveness in conversation to be more important compared to the conversational constraints of concerns for others’ feelings, non-imposition, and concern about disapproval, possibly aligning with IFL as it follows a more typical sentence structure used to describe other groups (Andrews et al., 2022). Conversely, individuals with an interdependent self-construal rated avoiding negative evaluations and hurting people’s feelings as most important, which may align with PFL as it is thought to be more inclusive and therefore potentially less offensive (Andrews et al., 2022; Kim et al., 1996). The parallels between language type and self-construal motivated this study’s investigation into the role of internal and dispositional factors, such as self-construal, in influencing identity perception and language preferences for disability.

The Big Five personality traits represent the most common scientific framework used to assess adult personality and includes openness, conscientiousness, extraversion, agreeableness, and neuroticism (John & Srivastava, 1999). Openness is one’s willingness to explore new ideas, creativity, and curiosity; conscientiousness addresses self-control; extraversion is one’s desire for social interaction; agreeableness concerns kindness and cooperation with others, and neuroticism measures irritability and moodiness. The Big Five are often used to explain individual differences in human tendencies. Melchers et al. (2016) found empathy to be predicted by agreeableness and conscientiousness, which are associated with following social norms (Geeraert et al., 2019). More specific to disability, Himmelberger et al. (2022) investigated whether the Big Five can predict attitude valence towards individuals with intellectual disabilities and found that openness and agreeableness were significant predictors. Radlińska et al. (2025) also found the Big Five to be correlated with attitude valence towards people with physical disabilities, where openness, agreeableness, and extraversion were the strongest predictors; however, the effect was small. Therefore, the Big Five personality traits serve as another possible internal factor for this study’s exploration of the elements influencing the perception of disability and language preferences.

Research on gender differences in language use has been mixed. Some studies have found gender differences, such as Haas (1979), who found that women used more polite and supportive language than men, and Mulac et al. (2001), who found that men used more direct language than women. However, other researchers, such as Weatherall (2002), argue that conclusions about gendered language differences are too broad and based on small text samples. If gender differences in language are present, it is possible that women may prefer PFL as it is perceived as inclusive, whereas men may prefer IFL because it is more direct. Kamenetsky and Sadowski (2020) found no difference between men’s and women’s perceptions of disability-related charity advertisements, regardless of language framing used, suggesting that gender does not impact how language shifts perceptions. Thus, the influence of language type on perceptions of PWDs may be consistent across genders.

Disability language can be explored at the intersecting point of linguistic difference, individual difference, and social change. At the linguistic level, the syntactic differences between PFL and IFL motivated investigation into how identity framing can modulate cognition related to disability. At the individual level, dispositional and demographic characteristics may contribute to differences in how people form concepts, judgments, and preferences about disability identity. At the broader level, disability language occurs within socially and institutionally developed linguistic norms, including professional guidelines, advocacy, disability community preferences, and sociocultural attitudes. The present study directly examined linguistic- and individual-level differences by testing PFL versus IFL framing and measuring demographic and dispositional factors. While social and linguistic norms were not directly measured by the present study, they are considered as crucial to the broader theoretical context for interpreting patterns of language preferences and observer judgments of disability-related identities.

### Purpose

The present study aimed to investigate how disability identity is conceptualized and linguistically described relative to non-disability-related identities. More specifically, the present study investigated the following confirmatory questions: (1) Does language framing, i.e., presenting an identity with PFL or IFL, influence the perception of how central an identity is to another’s sense of self? (2) Do conversational language preferences, specifically the use of PFL or IFL, vary depending on the identity type being referenced and whether or not this identity is a disability-related identity?

Despite mixed findings on disability and language, we hypothesized that language framing would influence perceived identity centrality because of the social emphasis on bias-free language coupled with linguistic support for the effects of language on perceptions of disability- and non-disability-related characteristics (Andrews et al., 2022; Granello & Gibbs, 2016; Karimi et al., 2019; Kelly et al., 2010; Weber, 2024). For language preferences, we hypothesized that PFL would be preferred for disability-related and IFL for non-disability-related identities because inclusive disability language guidelines often emphasize PFL, while IFL is typically the linguistic structure for referencing other identity types (Andrews et al., 2022; APA, 2024).

The connection between demographic and dispositional factors on disability language remains under-investigated with conflicting results; therefore, the following exploratory research questions were investigated: Does gender influence perceptions of identity centrality and preferences for disability language use? Can dispositional factors such as the Big Five personality traits, self-construal, and attitudes towards PWDs predict perceived identity centrality or language preferences? Finally, due to the present study’s focus on disability and the researchers prioritizing a diverse sample that included disabled individuals, it also explored whether disability status influences perception of disability and language preferences in order to further understand the influence of individual differences on disability perception and language preferences. By investigating both confirmatory and exploratory questions, this study sought to contribute to empirical research regarding the role that PFL and IFL play in shaping identity perception and identity-specific language choices.

## 2. Method

The present research is a quantitative study consisting of four sections: (a) a demographics questionnaire, (b) an Identity Centrality Perception Task, where participants indicated how central they perceived an identity marker to be to a target’s personal identity, (c) a preference task, where participants indicated the type of language (PFL, IFL) they would use in conversation when referring to a specific identity, or whether they have no preference (Either), and (d) dispositional surveys. The study was completed online by emerging adults recruited through either social media or a first-year psychology subject pool. The study was approved by the University of Toronto Research Ethics Board.

### 2.1. Participants

The study recruited 318 participants. Inclusion criteria required participants to be between 18 and 30 years of age in order to capture the language preferences and perceptions of emerging adults. This inclusion criterion was designed to reduce age-related confounds and focus on an age range characterized by attitude formation and linguistic change, making this group highly relevant to the present study (Arnett, 2000; Bigham, 2012). Exclusion criteria for responses required full or almost full completion of the two tasks (though up to two missing responses were accepted) and full completion of the dispositional scales to ensure a valid measure of the construct. This resulted in the exclusion of 80 individuals who did not meet the requirements. Thus, the final sample consisted of 238 participants (159 female, 60 male, and 19 other). Age ranged from 18 to 30 (*M* = 21.21, *SD* = 3.15). A small portion of the participants (16%) identified as disabled, whereas the majority did not (79%), or preferred not to answer (5%). The sample was ethnically diverse. 40% identified as White, 29% Asian, 8% Middle Eastern/North African/Southwest Asian, 3% Black, 1% Latino/a/x, 1% Indigenous and 18% identified as Other/Multiracial or preferred not to answer.

Participants were recruited through either social media or the University of Toronto Mississauga’s Department of Psychological & Brain Sciences undergraduate subject pool consisting of first- and second-year students. Participants from social media were recruited through virtual study flyer postings on various social media platforms through personal, institutional, student life, and student group accounts, as well as through department-wide student mailing lists, targeting a university-aged population that aligned with this study’s inclusion criteria. Researchers also used snowball sampling, encouraging social media users to share the study flyer with others to reach a broader and more diverse sample.

Participants recruited through the undergraduate subject pool received course credit for completing the study. Social media recruitments were provided with the option to enter a raffle for a gift card.

### 2.2. Measures

#### 2.2.1. Demographic Measures

Demographic information was collected using a modified version of the University of Toronto (2025) Equity Census. It collected basic demographic information such as age, gender, race, and disability status. This census is typically used by the University of Toronto to collect demographic information about its student population, so this study’s use of the census allowed researchers to effectively capture relevant identity-related information aligned with the diversity of this study’s university-aged student participants.

#### 2.2.2. Identity Centrality Perception Task

Participants were asked to indicate the level of perceived importance of 34 disability- or non-disability-related identities to a target’s sense of self. Disability-related identities were categorized into General Disability identities (e.g., disabled, neurodivergent), Sensory Disabilities (e.g., blind), Physical Disabilities, (e.g., paraplegic) Mental Illnesses (e.g., depressed), Neurological Disabilities (e.g., autistic), and Chronic Illnesses (e.g., diabetic). Non-disability-related were categorized into General-Non-Disability identities (e.g., queer, religious), Race (e.g., Black), Sexual Orientation (e.g., gay), and Religion (e.g., Hindu).

The disability-related categories were modified from the disability types outlined by Statistics Canada (2023b), to ensure a validated and broad set of disabilities were examined. When available, the labels used for disability-related stimuli were based on the APA style guide or on medical terminology (APA, 2024). However, it was important that the disabilities tested should follow strict linguistic framing (i.e., person-first or identity-first phrase structures) to avoid introducing confounding syntactic variations. Therefore, when medical or APA disability labels did not align with the study’s linguistic structure, colloquial terms were used instead (e.g., alcoholic). Non-disability-related categories included identity types that, colloquially, tend to appear with IFL framing. In this study, these non-disability-related categories serve as stimuli to compare against disability-related categories, which, based on the literature, are more varied in how their labels are referenced and how these identities are perceived by an observer. We recognize that colloquial, as well as medical terms can have negative or stigmatizing connotations; because of this, participants were provided with distress resources during the debriefing process.

Each broad identity category consisted of two to four specific identities to represent the group, providing a total of 34 unique identities. Table 1 presents the full list of the identity items. The General Disability category included broader, over-arching labels that tend to be used to describe a group of disabilities with similar features.

Participants were presented with a sentence with either PFL or IFL framing consisting of the target’s identity and a behaviour they are engaging in. Participants were then asked to rate how central they believe that identity is to the person being described using a 5-point Likert scale where 1 = not at all important and 5 = extremely important. Participants were randomly assigned either PFL or IFL framing for each of the 34 identities. For example, participants were presented with either “A person who is anxious stirs a pot.” or “An anxious person stirs a pot.” and asked to rate “How important is being anxious to who the person is?” This was repeated for each identity. Table 2 presents a full list of the identity phrases used.

The Identity Centrality Perception Task was modified from the noun perception task used by Weber (2024), which contained a single-item question that measured participants’ observer judgments of another person’s characteristics. The development of this one-item measure relates to the present study’s investigation of participants’ perceptions. Similar to the observer judgments measured in Weber (2024), rather than making self-reported judgments about their own identities, participants in the present study responded with how central they perceived an identity marker to be for a target other. The Identity Centrality Perception Task thus captures participants’ intuitive inferences about others based on limited information about their disability or non-disability categories.

#### 2.2.3. Language Preference Task

Language preferences were determined using a non-forced choice task where participants were presented with each of the 34 identities (see Table 1), one at a time, and asked to indicate whether they would use PFL, IFL, or Either, when referencing someone with this identity in conversation. The order the identities were presented was randomly assigned. The order in which the option to select PFL or IFL varied, while Either always remained the third option. For example, participants were asked “Please indicate which phrase you would most likely use in conversation when referring to this person or whether you would use either phrase equally: 1. Paraplegic person 2. Person who is paraplegic 3. Use each phrase equally”.

#### 2.2.4. Dispositional Measures

Dispositional information was collected using three preexisting questionnaires. (a) The John and Srivastava (1999) Big Five Personality Scale consisted of 44-items ranked on a 5-point Likert scale, where participants indicated how strongly they agreed with statements covering the five major dimensions of adult personality: openness, conscientiousness, extroversion, agreeableness, and neuroticism. (b) The Singelis (1994) Self-Construal—a 30-item questionnaire ranked on a 7-point Likert scale to determine one’s level of interdependence and independence of self-construal by having participants indicate their agreement with statements about a variety of feelings and behaviours in various situations. (c) The Myong et al. (2021) Attitudes and Perspectives toward Persons with Disabilities scale (APPD) determined valence of attitudes towards PWDs using a 14-item questionnaire where participants ranked their agreement with statements about PWDs on a 5-point Likert scale.

### 2.3. Procedure

The study was completed online in approximately 30 minutes. After providing informed consent and demographic information, participants completed the Identity Centrality Perception Task, followed by the preference task and the dispositional measures. Participants were thanked and debriefed.

Python (version 3.14.0) was used for data analysis. Data management was conducted using pandas (version 2.3.3), NumPy (version 2.3.4), SciPy (version 1.17.1), statsmodels (version 0.14.6), and Pingouin (version 0.6.0).

## 3. Results

### 3.1. Identity Centrality Perception Task

Data collected from the Identity Centrality Perception Task satisfied the relevant assumption checks required for analysis. A 2 × 2 repeated-measures ANOVA, including the within-subjects variables of Language Type (PFL, IFL) and Identity Type (disability, non-disability), was conducted on participants’ perceived centrality scores for the perception task. Scores were computed by averaging scores for disability and non-disability identities for both PFL and IFL, providing each participant four unique perceived centrality scores for perceptions of disability and non-disability identities separated by PFL and IFL. A significant main effect of Identity Type was obtained. Disability-related identities were rated as more important to a participant’s identity (M = 2.36, SD = 0.79) compared with non-disability-related identities (M = 1.76, SD = 0.88), *F*(1, 237) = 200.48, *p* < 0.001, *d* = 0.918. There was no significant main effect of Language Type, *F*(1, 237) = 1.29, *p* > 0.05, *d* = 0.074 and no significant interaction effect between Language Type and Identity Type, *F*(1, 237) = 1.86, *p* > 0.05, *d* = −0.088.

A 2 × 3 mixed effects ANOVA, including the within-subjects variables of Language Type (PFL, IFL) and between-subjects factors of Gender (male, female, other), was conducted on participant’s perceived centrality scores and revealed no significant main effect of Language Type, *F*(1, 235) = 0.08, *p* > 0.05, *η*^2^*p* < 0.001, Gender, *F*(2, 237) = 1.54, *p* > 0.05, *η*^2^*p* = 0.011, nor a significant interaction, *F*(2, 235) = 0.95, *p* > 0.05, *η*^2^*p* = 0.008.

A 2 × 2 mixed effects ANOVA was conducted on participant’s perceived centrality scores, including the within-subjects variables of Language Type (PFL, IFL) and between-subjects factors of Disability Status (disabled, non-disabled). Participants who did not disclose their disability status were not included in this analysis (*n* = 11). This analysis revealed no significant main effect of Language Type, *F*(1, 225) = 0.01, *p* > 0.05, *η*^2^*p* < 0.001, Disability Status, *F*(1, 225) = 0.01, *p* > 0.05, *η*^2^*p* < 0.001, nor a significant interaction, *F*(1, 225) = 2.47, *p* > 0.05, *η*^2^*p* = 0.011.

### 3.2. Preference Task

Participants’ overall language preferences for each identity category were determined by summing their coded preferences across each identity category (IFL = −1, PFL = +1, Either = 0). Positive totals indicated an overall PFL preference, negative totals an IFL preference, and scores of zero as Either preference. This data satisfied the relevant assumption checks for chi-squared analyses.

A chi-squared goodness-of-fit test was then conducted with the overall preference scores for each identity category. PFL was preferred for general disability, physical disability, mental illness, neurological disability, and chronic illness. IFL was preferred for sensory disabilities. IFL was also preferred for all non-disability-related identities. Table 3 presents the sample’s overall language preference breakdowns and chi-squared goodness-of-fit test results. To control the Family-wise Error Rate across the 10 chi-square tests, the Holm–Bonferroni correction was applied; all results remained statistically significant.

A chi-squared test of independence investigated the association between gender and overall language preference for disability-related identities. Overall disability-related language preference scores were computed by totaling each participant’s scores for all disability-related identities using the scoring method above. The relationship between gender and language preference was statistically significant, *χ*^2^(4, 238) = 15.82, *Cramér’s V* = 0.13, *p* < 0.01. Female participants were more likely to have an overall PFL preference than male participants, whereas male participants were more likely to have an overall IFL preference than female participants. Due to the small sample size (*n* = 19) of participants not identifying as male or female, no firm comparative conclusions were drawn regarding their language preferences.

A chi-square test of independence examined the association between participants self-reported disability status (i.e., disabled or non-disabled) and overall disability-related language preferences for disability-related identities. The association was not statistically significant *χ*^2^(4, 238) = 1.57, *Cramér’s V* = 0.04, *p* = 0.814. Thus, the data did not provide evidence that overall language preferences for referencing disability-related identities differed between disabled and non-disabled participants. The sample of participants who preferred not to disclose their disability status was small (*n* = 11); therefore, no firm comparative conclusions were drawn regarding this group’s language preferences.

### 3.3. Dispositional Predictors

#### 3.3.1. Reliability Estimates for Dispositional Predictors

Internal consistency was assessed for all measures in the present sample using Cronbach’s alpha and are as follows. The Big Five subscales: Extraversion, α = 0.87; Agreeableness, α = 0.77; Conscientiousness, α = 0.78; Neuroticism, α = 0.83; and Openness, α = 0.70. The Independent and Interdependent Self-Construal subscales, α = 0.78 and α = 0.76 respectively. The APPD, α = 0.84.

#### 3.3.2. Predicting Perception of Disability Identity Centrality

An ordinary least squares regression was conducted to examine whether the Big Five, self-construal, and attitudes towards PWDs predicted participants’ perceived identity centrality scores for both disability and non-disability-related identities. Two models were run to test the predictors against both Language Types (PFL, IFL). The Bonferroni correction was applied to account for running multiple regressions, adjusting the significance threshold from 0.05 to 0.025 to control for Type I error risk. The adjusted significance threshold was applied to the overall regression models; however, within the significant models, individual predictors were analyzed using unadjusted p-values.

The PFL model showed weak and nonsignificant results, *R*^2^ = 0.023, *F*(8, 229) = 1.70, *p* = 0.10, broadly suggesting that dispositional factors are not predictors of the perception of disability identity centrality for PFL-framed items.

The IFL model also showed weak but significant results, *R*^2^ = 0.058, *F*(8, 229) = 2.83, *p* < 0.05, broadly suggesting that dispositional factors may influence the perception of disability identity centrality when IFL-framed items were presented. The most significant predictor was attitudes toward PWDs, *b* = 0.03, *SE* = 0.007, *p* < 0.001, demonstrating that more negative attitudes toward disability may have increased the likelihood of a higher perceived importance for IFL-framed disability identities. Independent self-construal was another significant predictor for the IFL model only, *b* = 0.20, *SE* = 0.10, *p* < 0.05, demonstrating that independence may have increased the likelihood of a higher perceived centrality for IFL-framed disability identities.

#### 3.3.3. Predicting Preferences of Disability Language

A multinomial logistic regression was conducted to examine whether the Big Five, self-construal, and attitudes towards PWDs predicted participants’ overall disability-related language preferences (PFL, IFL, Either) for disability-related identities. An overall PFL preference was used as the reference category. The model was statistically significant, likelihood-ratio *χ*^2^(16, 238) = 36.42, *p* < 0.01, pseudo-*R*^2^ = 0.089, suggesting weak explanatory power.

PFL vs. IFL. Higher agreeableness was associated with higher odds of preferring PFL than IFL, *OR* = 1.52, 95% CI [1.05, 2.17], *p* < 0.05. More negative attitudes towards PWDs (higher scores) were associated with greater odds of preferring IFL to PFL, *OR* = 0.56, 95% CI [0.40, 0.77], *p* < 0.001 All other predictors were nonsignificant.

PFL vs. Either. Higher independent self-construal was associated with lower odds of preferring PFL rather than having no language preference, *OR* = 0.44, 95% CI [0.21, 0.93], *p* < 0.05. However, this result should be interpreted cautiously because it was based on a small portion of participants who exhibited no language preference (*n* = 16).

Since the sample included participants from several recruitment sources these groups may have been exposed to slightly different messaging about disability language. a chi-square test of independence was performed to examine whether any differences were evident. Small but significant differences in overall disability-related language preferences between the two groups *χ*^2^(2, 238) = 6.04, Cramér’s *V* = 0.16, *p* = 0.49 showed that undergraduate participants were more likely to express overall PFL preferences, whereas social-media participants were more likely to express IFL or Either preferences.

## 4. Discussion

The present study aimed to investigate differences in how disability- and non-disability-related identities are conceptualized by measuring whether participants’ perception of how central an identity is to another’s sense of self is influenced by PFL or IFL framing.

Adherence to language trends were investigated by measuring language preferences for various disability- and non-disability-related identities. Potential influences on perception and language use were investigated by seeing if language preferences and perception of identity centrality could be predicted by dispositional factors as well as differences between genders and disability status. By doing so, this study aimed to provide an interpretation of how dispositional, demographic, and broader social factors influence how disability is understood and spoken about. Results showed that identity type, specifically disability-related identities, were perceived to be more central to another’s self-concept than non-disability-related identities, while language framing (PFL vs IFL) and gender and disability status did not significantly influence perception. Analysis of language preferences indicated that PFL was preferred for all disability-related identities, apart from sensory disabilities, for which IFL was preferred, while IFL was preferred for all non-disability-related identities. Gender differences in language preference were present, where female participants preferred PFL and male participants preferred IFL. Disabled and non-disabled participants expressed similar overall preferences for disability language. Dispositional factors were significant but weak predictors of perception and language preference. The following sections contextualize and explain the findings and discuss their implications.

### 4.1. Identity Centrality Perception Task

Disability-related markers were generally rated by participants as more central to another’s identity than non-disability-related markers; Language Type (PFL/IFL) did not affect perceived centrality ratings. These findings did not align with the hypothesis that language framing would influence perception. However, the lack of a significant Language Type effect is supported by prior research showing that disability language framing had minimal effects on evoking more positive or negative attitudes toward disability (Kamenetsky & Sadowski, 2020; Patterson & Witten, 1987). The results of the repeated-measures ANOVA reveal that perceptions of identity centrality were primarily driven by identity type, suggesting that PFL vs IFL manipulations may play a limited role in shaping how strongly disability is perceived as part of another’s self-concept.

Disability identities were perceived to be more important to another’s sense of self than non-disability identities, such as race, religion, and sexual orientation, suggesting that participants may have relied more on their conceptualization of disability as an identity category instead of linguistic cues when making social judgements. A possible explanation for the Identity Type effect is that disability is perceived as uniquely impacting everyday functioning compared to non-disability-related identities, making disability a more salient marker of personal identity and self-concept compared to other social categories (Marks, 1999; Murugami, 2009). As Murugami (2009) found, the self-formation of non-disabled individuals often establishes exclusionary processes that delineate impairment as exceptional, which may perpetuate the idea that disability is a fixed and separate identity warranting greater perceived centrality and importance for a disabled person’s sense of self. Overall, these results suggest a systematic difference in how people make judgements of disability in relation to a person’s identity, highlighting that disability may be perceived as a more essential and salient marker of an individual’s self-concept compared to non-disability markers.

Linguistic structure was observed to play a minimal role in shaping perceptions of disability identity, countering prior research indicating greater encoding strength for post-modified nouns, i.e., stronger conceptual association of “person” with their identity marker for PFL framing (Karimi et al., 2019; Weber, 2024). For disability language and identity markers, perceptions of identity centrality were not influenced by the syntactic framing of the noun in the same way that standard modifying adjectives were. Although this task’s distinction between PFL and IFL did not yield significant language effects on the perception of disability identity, language appears to be able to encode negative stereotypes and stigma, such as the idea that disability is wholly identity-constitutive in deficit-oriented language that frames disability as a fixed impairment (Andrews et al., 2022; Dunn & Andrews, 2015; Marks, 1999).

This task also found no significant difference between male and female participants’ ratings of perceived identity centrality for PFL- or IFL-framed identities, supporting prior research by Kamenetsky and Sadowski (2020).

This task also found no significant difference between self-identified disabled and non-disabled participants’ ratings of perceived identity centrality for PFL- or IFL-framed identities, suggesting that individual disability experience did not meaningfully influence observer judgments of disability identity centrality. Thus, these perceptions may possibly reflect broader social conceptualizations of disability that are shared across disabled and non-disabled participants, rather than being primarily shaped by lived disability experience. Furthermore, the disability statuses of participants do not reflect the true population-level diversity of disability because of the study’s sample containing university-aged students, which likely further minimized the influence of personal disability experience on perceived identity centrality.

Social judgements of identity centrality were consistent across gender and disability status in the sample and further supporting the notion that mental conceptualization and understanding of identity markers, rather than personal attributes, influence how individuals perceive disability identity centrality.

### 4.2. Preference Task

PFL was preferred for all disability-related identities except sensory ones, where IFL was favoured, whereas IFL was preferred for all non-disability-related identities. This result aligned with the hypothesis that PFL would be preferred for disability-related identities. However, results for sensory disabilities were an exception since IFL was the majority preference for these identities instead. These findings suggest that disability is discussed and perhaps understood differently than non-disability-related identities. This aligns with tendencies to perceive disability as a condition rather than a feature of human diversity (Marks, 1999). Disability is often framed as an internal deficit to be treated (Smart, 2009). Despite advocacy for PFL to shift the view from deficit to human difference, disability advocates argue that encouraging a distinct language type for disabilities has reinforced the notion that disability is different than other identity markers (Andrews et al., 2022; Brown, 2011; Gernsbacher, 2017).

Nonetheless, some participants had no preference for language type or even preferred IFL over PFL when referencing disability, particularly for sensory disabilities. A possible reason for this difference is that sensory disability communities, such as the Deaf community, reject PFL since they view deafness as not inherently negative or something that can be separated from one’s identity and, thus, should be placed at the forefront (Andrews et al., 2022; Dunn & Andrews, 2015). Similarly, the National Federation of the Blind (1993) rejected PFL, labelling it as implying negativity rather than promoting equality. This has since been reflected in language guidelines, such as the United Nations’ (2019) disability-inclusive language guidelines recommending IFL framing for sensory disabilities but not for other disability groups. Therefore, it is possible the language encouraged by these guidelines has become mainstream, as reflected by the sample’s preference for IFL for sensory disabilities.

It is also possible that participants preferred IFL for sensory disabilities because they were perceived differently than the other disability groups tested. Canton et al. (2023) grouped disability types into clusters based on perceived warmth, competence, and courage. Individuals with sensory disabilities fell into the cluster with the highest perceived competence compared to the other disabilities measured. Thus, IFL was possibly preferred for sensory disabilities because this group is perceived as more competent, creating less desire for participants to separate the individual from their disability.

The results of the preference task suggest that IFL is also gaining popularity perhaps due to criticisms of PFL, group-specific preferences, and beliefs about specific disability types. This shift is apparent when comparing the results of the present study to Lynch et al. (1994) who found that 60% of participants preferred to say, “person with a disability” whereas 7% preferred saying “disabled person”. In contrast, the present study found that 49% of participants preferred PFL whereas 30% preferred IFL for general disability labels. Arguably, using a different language structure when referencing disability reinforces the notion that disability is a fixed condition separate from other identities (Andrews et al., 2022; Dunn & Andrews, 2015; Marks, 1999). If shifts are being made to label disability as a group identity rather than an inherently negative diagnosis, as emphasized by the sociopolitical model and rooted in self-acceptance, IFL should be used when referencing disability in the same way that non-disability identities are (Andrews et al., 2013; Andrews et al., 2022; Smart, 2009). This influence may be shown by the proportion of participants who indicated they would use PFL or IFL interchangeably across both disability- and non-disability-related identities, and those who preferred IFL. However, preferences for PFL were consistently lower for non-disability-related than disability-related identities, indicating that PFL is much less associated with referencing non-disability-related identities.

Analyses on gender differences in language preference found that more women preferred PFL whereas more men preferred IFL. This finding is consistent with past research indicating that women tend to use more polite language, whereas men are more direct (Haas, 1979; Mulac et al., 2001).

No relationship between disability status and language preferences for disability-related identities was found, suggesting that disabled and non-disabled participants indicated similar overall preferences for referencing disabled individuals in conversation. This may be due to the sample primarily consisting of university students whose own disabilities, which were likely relatively mild, may not have aligned with the ones tested, so language preferences were less shaped by their own disability experience.

### 4.3. Dispositional Predictors

While the dispositional models were statistically significant, they had weak explanatory power, suggesting a minimal practical significance of disposition as a predictor of perceived disability identity centrality and language preferences. The regression analysis results suggest that internal personality or dispositional traits guiding how people perceive and speak about disability may play a minimal role in shaping judgments of disability identity. Possibly, though not assessed in this study, external social and linguistic norms may be more prominent influences.

Haller et al. (2006) provided support for this notion by showing that more inclusive language was used in major American media outlets following the US Disability Rights Movement, highlighting that broader institutions may guide how the public uses language and that policy and advocacy can make meaningful changes in language and perceptions of social groups. It is important to note, however, that some studies support the role of dispositional factors (see Radlińska et al., 2025). The latter study found that quality of contact with PWDs mediated the relationship between personality and attitudes. This observation suggests that while dispositions matter, external factors provide strong influences on perception of disability.

## 5. Implications

This study suggests that people perceive disability as different from other identities, as demonstrated by participants’ PFL preference for most disability identities and ratings of disability identities as more central to another’s sense of self. Differences in social judgments for disability versus non-disability identities may also be reflected in language preferences. While advocacy for PFL has been driven by the desire to emphasize a PWD’s humanity and reduce disability to merely indicating difference, our results show that disability is still perceived as salient, suggesting that encouraging PFL for referencing disability may not meet its intended goal of moving focus away from an individual’s disability status (Andrews et al., 2022; Gernsbacher, 2017; Kapitan, 2017).

The results suggest that subtle changes in language construction to describe disability may not be the most effective method to foster change in how disabilities are perceived. While participants showed preferences for language type that differed based on the identity described, PFL versus IFL framing did not actually influence how participants judged these identities. It is possible that language preferences are driven by linguistic trends surrounding what is believed to be the kindest way to describe people and their identities, rather than by empirical evidence concerning how different linguistic constructions for describing people influence the perception of their identities. The lack of influence that PFL or IFL manipulations have on this study’s measure of other identity perception, coupled with the lack of explanatory power of dispositional and demographic predictors, suggest that other factors, possibly linguistic norms, may serve as a more prominent driver of language preferences rather than individual characteristics or minor language changes.

The present findings may also highlight the possible influence of social norms on disability-related language preferences. Participants’ preferences for PFL, together with the limited influence of the dispositional factors examined, is consistent with the possibility that widely established language conventions may shape preferences more strongly than individual dispositions. PFL has been promoted through disability advocacy and inclusive professional and media language guides, making it a familiar convention for many young adults who grew up during its widespread adoption. Furthermore, the higher levels of IFL preferences in comparison to older research aligns with more recent IFL movements, again suggesting the possible impact of external trends on language. Although social norms were not directly measured, these findings suggest that other factors, such as large-scale social movements may contribute to broad changes in language use by establishing shared linguistic conventions.

However, beyond the PFL and IFL distinction, language may in fact influence how people perceive and conceptualize their environment, an observation supported by proponents of the SWH, as well as by research on the impact of negative disability terminology (Granello & Gibbs, 2016; Haller et al., 2006; Kamenetsky & Sadowski, 2020; Kelly et al., 2010; Muasya, 2023; Wolff & Holmes, 2010). If the present study had found PFL or IFL to significantly differ in their impact on the perception of disability, there may be reason to encourage language that promotes the view of disability as a feature of human diversity similar to other non-disability-related identities, rather than a negative impairment. However, when strictly comparing PFL to IFL, these syntactic differences did not show systematically different effects on disability identity perception, despite a tendency for participants to report using PFL for most disability-related identities. Thus, rather than prescribing specific language types deemed more appropriate than others, being respectful in communication and prioritizing the language preferences of disability groups may foster more meaningful change, ensuring the unique experiences of disability are considered in everyday language. This recommendation also implies that language alone will not improve the lived experience of people with disabilities and, as such, focus should shift toward meaningful actions.

## 6. Limitations and Directions for Future Research

Due to the breadth of variables investigated in the present study, there are some limitations that may be addressed by further research in this field.

First, the sample was primarily composed of female participants (67%); however, this is common among psychological research and is generally not viewed as a problem when at least 30 participants of the underrepresented group are included (*n_male_* = 60 [25%]; Clay, 2017, Hogg & Tanis, 2009). Second, only 16% of the sample identified as having a disability. Much of the research on disability language focuses on the experiences of disabled individuals, which the current sample did not address in depth. However, consistent with the social model of disability, the present study sought to understand social perceptions of disability from university-aged individuals in general rather than the specific perspectives of disabled individuals. To do this, a diverse sample was required, and having 16% of participants identifying as disabled is generally consistent with Canadian national survey estimates that approximately 20% of youth aged 15–24 have a disability (Statistics Canada, 2023a). Furthermore, analyses comparing the results of disabled and non-disabled participants were not statistically significant, suggesting that both groups responded to the tasks similarly and that, for this specific study, analyses that combined disabled and non-disabled participants were appropriate. Third, the slightly higher counts of PFL preferences among the undergraduate participants suggest that context (e.g., undergraduate students encountering to PFL in coursework) may shape language norms, and including multiple recruitment sources may increase the generalizability of our findings by capturing the preferences of young adults who are not necessarily university students. Future research could examine what factors contribute to different language preferences across these groups. When examining the results of the perception task between the participant pools, no significant differences between the groups were found, further suggesting that these cohort differences are minor.

Limitations surrounding the demographic items measured are also noted. Many factors have the potential to influence perceptions of and language preferences for disability, such as quality of contact with PWDs (see Radlińska et al., 2025). Apart from disability status, other disability-related traits were not measured. The researchers recognize that participants likely had a variety of experiences with and knowledge of disability which may have influenced their responses. However, the aim was for a broad sample of participants that encompassed a variety of different lived experiences and were limited in the number of factors they could feasibly measure and consider in analyses. Regardless, future researchers should aim to collect more disability-related data such as specific disability identities present in the sample and level of contact with disabled individuals or prior disability-related studies to see if these factors play a role in perception of disability and language preferences.

The researchers also acknowledge the limitations of the methodology used in the present study. The Identity Centrality Perception Task required judgements to be derived from a single rating item rather than a multi-item measure and as such are not ideal. Since participants were tasked with evaluating multiple identities presented with different linguistic constructions, a single rating allowed the design to remain feasible as they performed repeated brief judgments across different identity categories. Furthermore, during the analysis of perceived identity centrality scores, participant responses were aggregated across broad identity categories (i.e., disability- versus non-disability-related) rather than the narrower one-item scores for each identity label, yielding a more robust measure of perceived identity centrality. Prior studies investigating perceptions based on linguistic framing use similar one-item measures that specifically target responses about identity judgments (Kamenetsky & Sadowski, 2020; Weber, 2024). The goal of this perception task, rather than establishing a psychometric assessment of identity centrality, was to capture participants’ social judgements of disability and non-disability identity markers; thus, this study’s measure of perceived identity centrality should be interpreted as an informative account for the perception of how important a specific identity is to a target’s general “self.” Future research investigating identity centrality as a standardized construct would nevertheless benefit from developing a multi-item scale that better distinguishes between different dimensions of personal identity and self-concept.

The present study utilized strict and consistent wording when presenting identities (i.e., for PFL, “a(n) [identity] person,” and for IFL, “a person who is [identity]”) to ensure that only the word order differences between PFL and IFL framing were captured. This, however, limited the identities that could be included in the study. For example, cerebral palsy would not fit into the study’s language framing; IFL framing may have looked like “a person who *has* cerebral palsy” because “a person who *is* cerebral palsy” is grammatically incorrect. Therefore, identities that are typically presented with “who *has*” rather than “who *is*” were not included in the present study. Further studies should investigate perceptions and language preferences for other identities that did not fit into the present study’s linguistic framework.

Another element that the present study was unable to address is intersectionality of identities. In the social world, people are not experienced as isolated labels that describe their identity; rather, a blend of different characteristics contribute to a unique life experience that is not captured by the simple summing of individual identity words (Purdie-Vaughns & Eibach, 2008; Wickenden, 2023). The authors acknowledge that the findings of the present study do not extend to more nuanced cases of making judgments about multiple identity traits. Instead, the tasks in this study were used to explore participant’s identity perceptions and language preferences when a person is described by a single label, allowing researchers to isolate a single target identity while controlling for other linguistic elements. However, the authors also note that disability itself is a social category distinguished by functional differences and is often siloed into a separate category of identity, which often leads to the reduction of disabled people, regardless of intersecting identities, to their condition or disability labels (Marks, 1999; Wickenden, 2023). Although this experimental isolation was useful for examining the effects of identity and language type, it necessarily reduces the complexity with which identities are encountered in everyday social perception. Measuring all possible combinations of the many components that make up complex identities—while theoretically meaningful—may present significant research design difficulties. Future research may explore how perceptual and preference judgments are made in more naturalistic settings in which people are experienced as intersecting identities.

## 7. Conclusions

The results of this study demonstrate that disability may be conceptualized and described differently from non-disability-related identities. Additionally, internal factors were weakly influential on perception and language indicating that broader social factors may play a stronger role in shaping judgements about disability and language. Regarding the ongoing debate about best practices for disability language use, leading linguistic guidelines may benefit from considering and incorporating the perspectives of disability communities rather than providing prescriptive recommendations. By prioritizing respectful communication and uplifting the voices of disability communities, future empirical research in disability and psychology can move toward more person-centred approaches that reflect a nuanced and diverse understanding of disability identity, language, and social perceptions.

## Figures and Tables

**Table 1:** Individual identity items by category.

Category	Identity
Disability-related	
General disability	Disabled
	Sick
	Mentally ill
	Neurodivergent
Sensory disability	Blind
	Deaf
Physical disability	Paraplegic
	Physically disabled
Mental illness	Depressed
	Anorexic
	Anxious
	Alcoholic
Neurological disability	Autistic
	Intellectually disabled
	Dyslexic
	Epileptic
Chronic illness	Diabetic
	Asthmatic
	Arthritic
	Obese
Non-disability-related	
General non-disability	Religious
	Queer
Race	Black
	Asian
	White
	Indigenous
Sexual orientation	Gay
	Straight
	Bisexual
	Lesbian
Religion	Christian
	Jewish
	Muslim
	Hindu

**Table 2:** Identity Centrality Perception Task Stimuli.

Identity	Scenario	Language Framing	Presented Stimuli
Disability-related			
General Disability			
Disabled	Takes a picture	PFL	A person who is disabled takes a picture.
		IFL	A disabled person takes a picture.
Sick	Wipes the table	PFL	A person who is sick wipes the table.
		IFL	A sick person wipes the table.
Mentally ill	Prepares a meal	PFL	A person who is mentally ill prepares a meal.
		IFL	A mentally ill person prepares a meal.
Neurodivergent	Smells a flower	PFL	A person who is neurodivergent smells a flower.
		IFL	A neurodivergent person smells a flower.
Sensory Disability			
Blind	Buys a sweater	PFL	A person who is blind buys a sweater.
		IFL	A blind person buys a sweater.
Deaf	Greets a neighbour	PFL	A person who is deaf greets a neighbour.
		IFL	A deaf person greets a neighbour.
Physical Disability			
Paraplegic	Waits for the bus	PFL	A person who is paraplegic waits for the bus.
		IFL	A paraplegic person waits for the bus.
Physically disabled	Bakes a cake	PFL	A person who is physically disabled bakes a cake.
		IFL	A physically disabled person bakes a cake.
Mental Illness			
Depressed	Receives a gift	PFL	A person who is depressed receives a gift.
		IFL	A depressed person receives a gift.
Anorexic	Uses a phone	PFL	A person who is anorexic uses a phone.
		IFL	An anorexic person uses a phone.
Anxious	Stirs a pot	PFL	A person who is anxious stirs a pot.
		IFL	An anxious person stirs a pot.
Alcoholic	Sings a song	PFL	A person who is alcoholic sings a song.
		IFL	An alcoholic person sings a song.
Neurological Disability			
Autistic	Opens a box	PFL	A person who is autistic opens a box.
		IFL	An autistic person opens a box.
Intellectual disability	Plays a game	PFL	A person who is intellectually disabled plays a game.
		IFL	An intellectually disabled person plays a game.
Dyslexic	Pets a dog	PFL	A person who is dyslexic pets a dog.
		IFL	A dyslexic person pets a dog.
Epileptic	Joins a club	PFL	A person who is epileptic joins a club.
		IFL	An epileptic person joins a club.
Chronic illness			
Diabetic	Checks the mail	PFL	A person who is diabetic checks the mail.
		IFL	A diabetic person checks the mail.
Asthmatic	Goes to sleep	PFL	A person who is asthmatic goes to sleep.
		IFL	An asthmatic person goes to sleep.
Arthritic	Goes outside	PFL	A person who is arthritic goes outside.
		IFL	An arthritic person goes outside.
Obese	Waters a plant	PFL	A person who is obese waters a plant.
		IFL	An obese person waters a plan.
Non-disability-related			
General non-disability			
Religious	Goes to work	PFL	A person who is religious goes to work.
		IFL	A religious person goes to work.
Queer	Attends an event	PFL	A person who is queer attends an event.
		IFL	A queer person attends an event.
Race			
Black	Makes a sandwich	PFL	A person who is Black makes a sandwich
		IFL	A Black person makes a sandwich
Asian	Takes a shower	PFL	A person who is Asian takes a shower
		IFL	An Asian person takes a shower
White	Cleans the dishes	PFL	A person who is White cleans the dishes
		IFL	A White person cleans the dishes
Indigenous	Writes a letter	PFL	A person who is Indigenous writes a letter
		IFL	An Indigenous person writes a letter
Sexual orientation			
Gay	Schedules a meeting	PFL	A person who is gay schedules a meeting
		IFL	A gay person schedules a meeting.
Straight	Packs a bag	PFL	A person who is straight packs a bag.
		IFL	A straight person packs a bag.
Bisexual	Meets a friend	PFL	A person who is bisexual meets a friend.
		IFL	A bisexual person meets a friend.
Lesbian	Goes to the store	PFL	A person who is lesbian goes to the store.
		IFL	A lesbian person goes to the store.
Religion			
Christian	Eats a snack	PFL	A person who is Christian eats a snack.
		IFL	A Christian person eats a snack.
Jewish	Drinks tea	PFL	A person who is Jewish drinks tea.
		IFL	A Jewish person drinks tea.
Muslim	Leaves the house	PFL	A person who is Muslim leaves the house.
		IFL	A Muslim person leaves the house.
Hindu	Puts on a shirt	PFL	A person who is Hindu puts on a shirt.
		IFL	A Hindu person puts on a shirt.

**Table 3:** Overall language preferences for identity categories and chi-squared goodness-of-fit.

Identity Category	Language Preference Breakdown			*N*	*χ* ^2^	*Cramér’s V*	*df*
	PFL	IFL	Either				
General disability	**48.74%**	29.83%	21.43%	238	27.94 **	0.24	2
Sensory disability	26.47%	**42.86%**	30.67%	238	10.34 *	0.15	2
Physical disability	**40.34%**	21.43%	38.24%	238	15.34 **	0.18	2
Mental illness	**46.64%**	33.19%	20.17%	238	25.02 **	0.23	2
Neurological disability	**53.78%**	26.47%	19.75%	238	46.39 **	0.31	2
Chronic illness	**55.88%**	23.11%	21.01%	238	54.61 **	0.34	2
General non-disability	14.71%	**53.36%**	31.93%	238	53.55 **	0.34	2
Race	13.45%	**74.37%**	12.18%	238	180.41 **	0.62	2
Sexual orientation	22.27%	**61.34%**	16.39%	238	85.27 **	0.42	2
Religion	21.85%	**54.62%**	23.53%	238	48.64 **	0.32	2

Note. Boldfaced indicates overall language type preferred by sample, * indicates *p* < 0.01., ** indicates *p* < 0.001. All tests remained significant after Holm–Bonferroni correction (*p_Holm_* ≤ 0.006).

## Data Availability

The data presented in this study are available on request from the corresponding author.
